# Causal Responsibility and Counterfactuals

**DOI:** 10.1111/cogs.12054

**Published:** 2013-07-15

**Authors:** David A Lagnado, Tobias Gerstenberg, Ro'i Zultan

**Affiliations:** a Department of Cognitive, Perceptual & Brain Science, University College London; b Department of Economics, Ben-Gurion University of the Negev

**Keywords:** Causality, Counterfactuals, Responsibility, Attribution, Shared responsibility, Pivotality, Criticality

## Abstract

How do people attribute responsibility in situations where the contributions of multiple agents combine to produce a joint outcome? The prevalence of over-determination in such cases makes this a difficult problem for counterfactual theories of causal responsibility. In this article, we explore a general framework for assigning responsibility in multiple agent contexts. We draw on the structural model account of actual causation (e.g., Halpern & Pearl, 2005) and its extension to responsibility judgments (Chockler & Halpern, 2004). We review the main theoretical and empirical issues that arise from this literature and propose a novel model of intuitive judgments of responsibility. This model is a function of both pivotality (whether an agent made a difference to the outcome) and criticality (how important the agent is perceived to be for the outcome, before any actions are taken). The model explains empirical results from previous studies and is supported by a new experiment that manipulates both pivotality and criticality. We also discuss possible extensions of this model to deal with a broader range of causal situations. Overall, our approach emphasizes the close interrelations between causality, counterfactuals, and responsibility attributions.

## 1. Introduction

Three police marksmen killed a barrister during a siege at his house in London. The barrister was drunk and brandishing a shotgun. Earlier on that afternoon he had fired shots out of the window; a 5-hour siege ensued, with a large number of police surrounding the house. During the siege police negotiators spoke to the barrister. He was distressed and suicidal, but the negotiators were unable to persuade him to surrender. After holding up a suicide note at the window, the barrister aimed his rifle at the police. He was shot dead in a volley of bullets. The post-mortem revealed that the barrister died from three critical gunshot wounds, to the brain, heart, and liver, each of which “would have been capable of causing death in their own right.” Moreover, the post-mortem stated that “the likely survivability of the brain wound, heart wound, or liver wound would have been very low, even if one was artificially in an intensive care unit at the time it happened.” An inquest ruled that each of the police shots was “lawfully fired”; that is, the police used reasonable force in defense of themselves or others. However, the jury criticized the police handling of the siege. They argued that the police should have considered letting the barrister's wife, or his close friend, speak to him during the siege; that the police gave insufficient weight to the fact the barrister was an alcoholic, who was drunk and therefore vulnerable; and that the police command structure was inadequate. Despite these criticisms, the jury decided that none of these shortcomings contributed to the barrister's death.^1^

This tragic case illustrates the close links between causation, counterfactuals, and responsibility attributions. For example, the jury in the inquest had to decide whether the police handling of the siege contributed to the barrister's death. Presumably, this required considering counterfactual situations where the police did not make these mistakes, and determining whether the fatal shooting would still have resulted. For instance, what would have happened if the barrister's wife had been allowed to contact him earlier in the siege? Would she have been able to pacify her husband? This is a crucial but difficult question. The jury ruled that it would not have made a difference to the final outcome, whereas the family and friends of the barrister argued the opposite. Indeed, during the siege the police decided that allowing the wife to speak to the barrister might endanger her life, or lead to a hostage situation, or accelerate the barrister's suicide by allowing him to say good-bye to his wife. These are a complex set of issues that hinge upon both causal and counterfactual thinking.

The case also provides a real-world example of over-determination, which plays a central role in philosophical discussions of causality (Collins, Hall, & Paul, 2004; Moore, 2009) and is seen as a major problem for standard counterfactual theories of causation. The official causes of death, as stated in the post-mortem, were the three gunshot wounds. This conclusion was supported by two counterfactual claims, both of which go beyond the kind of counterfactual analysis offered by the standard theory due to Lewis (1973, 1986). Indeed, these more sophisticated counterfactuals fit well with a modified counterfactual account (Halpern & Pearl, 2005) that will be explored in this article. Finally, the case also raises issues of responsibility and blame. The police marksmen were found to be causally responsible for the barrister's death, but they were not held legally responsible, because they carried out their actions in reasonable self-defense (or defense of others). This shows that attributions of causal responsibility do not automatically translate to attributions of blame or legal responsibility (Shaver, 1985). Moreover, the judgment about legal responsibility here also seems to invoke counterfactual thinking—a marksman's shot was reasonable if, without it, the victim *would* have endangered the lives of the marksmen or their colleagues.

In this article, we will explore a general framework for assigning causal responsibility in multiple agent contexts. We draw on the structural model account of causality (Pearl, /2009^2000^; Halpern & Pearl, 2005; Woodward, 2003), and its extension to responsibility judgments (Chockler & Halpern, 2004). Building on this theoretical work, we will propose a novel model of intuitive judgments of responsibility that is a function of both pivotality (whether an agent made a difference to the outcome) and criticality (how important the agent is perceived to be for the outcome, before any actions are taken). This model explains empirical results from previous studies (e.g., Gerstenberg & Lagnado, 2010; Zultan, Gerstenberg, & Lagnado, 2012) and is supported by a new experiment that manipulates both pivotality and criticality. We will also discuss possible extensions of this model to deal with a broader range of causal situations. Overall, our approach emphasizes the close interrelations between causality, counterfactuals, and responsibility attributions.

### 1.1. Legal aspects of responsibility

Although our focus is on intuitive judgments of causal responsibility, it is instructive to consider some aspects of the legal conception of responsibility. A key idea in contemporary theories of legal responsibility (e.g., Cane, 2002; Hart, 2008; Honoré, 1999) is that the function of responsibility is both backward and forward looking. *Retrospective* (or historical) responsibility concerns issues of accountability for what has actually happened, and is the basis for blame and punishment. *Prospective* responsibility concerns duties and obligations for future events: the prevention of bad outcomes and the production of good outcomes (Cane, 2002). An example is Hart's notion of role responsibility, where someone takes responsibility for a specific set of tasks. The distinction is readily applied to the siege case. One can consider the retrospective responsibility that the marksmen, and the police command more generally, actually bear for the barrister's death. But one can also consider their prospective responsibilities (before the barrister was shot); namely, to safeguard the lives of bystanders, relatives, and the police, and to maintain law and order.

Although legal commentaries tend to focus on retrospective responsibility, Cane (2002) argues that prospective responsibility is equally important. The legal function of responsibility attributions is not simply to punish bad behavior in the past, but to prevent it occurring in the future. Indeed Cane states: “historic responsibility finds its role and meaning only in responding to nonfulfillment of prospective responsibilities” (2002, p. 35).

It is natural to extend this argument to the function of responsibility attributions in everyday (non-legal) contexts. Presumably, people's intuitive judgments should also be sensitive to both forward- and backward-looking concerns. In parallel with the legal literature, however, formal and psychological models of responsibility mainly focus on retrospective evaluations. This article will likewise build first upon accounts of retrospective responsibility. However, as discussed later in this article, the notion of prospective responsibility emerges as an important factor in people's intuitive attributions, even when they are making purely retrospective judgments.

### 1.2. Counterfactual models of causation

The analysis of causality has been an ongoing problem in philosophy and psychology ever since Hume (/1975^1748^). There have been many attempts to define causation in terms of non-causal concepts such as regularities (Mackie, 1974), probabilities (Suppes, 1970), counterfactuals (Lewis, 1986), or physical processes (Dowe, 2000). None of these reductionist programs has achieved any consensus, and the debate continues (Beebee, Hitchcock, & Menzies, 2009). The advent of the structural model framework (Pearl, /2009^2000^; Spirtes, Glymour, & Scheines, 2000; Woodward, 2003) has sharpened many of the debating points and introduced a novel conception of causality in terms of interventions and counterfactuals. In short, the structural model approach proposes that A causes B if and only if there are potential interventions on A that would lead to changes in B (or changes in the probability distribution over the value of B). Here, the causal relata are variables, and causal relations between variables are represented by modifiable structural equations (see Woodward, 2003, for an accessible account).

A key feature of this framework is that it is non-reductionist. Causal relations are defined in terms of potential interventions (which themselves appeal to causal assumptions). We see this as an advance rather than a shortcoming, especially with regard to psychological theorizing. Rather than search for a reductive definition, causality is accepted as a primitive notion, and the causal model framework formalizes and clarifies the relations between causal models, probability, interventions, and counterfactuals (see Pearl, /2009^2000^; Sloman, 2005). This avoids the problem, particularly acute for counterfactual theories, of grounding or justifying counterfactuals without appeal to causal knowledge (cf Edgington, 2011; Woodward, 2011). The structural account allows for close interrelations between causal and counterfactual claims, while accepting that the former are not reducible to the latter.

#### 1.2.1. General versus actual causation

Philosophical analyses of causation distinguish between *general* and *particular* causal claims. A general claim—such as “smoking causes cancer” or “shootings cause deaths”—refers to classes of events or properties, and represents a generic causal relation/mechanism between these classes, without specifying a particular instantiation of the relation. In contrast, a particular causal claim refers to an actual case in which the causal relation holds. For example, that Joe's smoking caused his cancer, or that marksman A caused the victim's death.

General causal claims are central to scientific enquiry and play a major role in prediction and control (Pearl, /2009^2000^; Woodward, 2003). Thus, causal knowledge about ballistics and the nature of human physiology allows us to predict the likely consequences of shooting someone at close range. However, general causal beliefs also serve as the background knowledge base needed to address questions of actual causation. To approach the question of whether marksman A's shot killed the victim, we draw on generic causal knowledge and assumptions, even though this knowledge is not by itself sufficient to deliver our final judgment.

Actual causal claims also play a role in science, especially in the interpretation of empirical findings to support more general causal theories. Some branches of science also concern actual causation directly such as theories about the big bang, or the evolutionary history of species. In addition, actual causal claims are critical in many practical and social contexts, in particular those involving explanation and responsibility attribution (e.g., law, history, politics, everyday social interactions). Despite this ubiquity, there is no generally agreed-upon definition of actual causation, and the various attempts in the philosophical literature all suffer from difficulties (Beebee et al., 2009). An attractive aspect of the structural model approach is that it offers a unifying framework for both general and actual causation (Halpern & Pearl, 2005; Pearl, /2009^2000^). It also maintains a close relation between causation and counterfactuals at both levels. Thus, both general and actual causal claims imply, and are supported by, counterfactuals. The relation is not, however, reductive or definitional.

#### 1.2.2. The standard counterfactual model

On the standard counterfactual model of causation (Lewis, 1973, 1986), “actual” causal claims are analyzed in terms of counterfactuals. Roughly, *c* causes *e* if and only if: (a) *c* and *e* are both true, (b) if *c* had not occurred, then *e* would not have occurred. For example, when enquiring whether Joe's smoking caused his cancer, one needs to consider whether Joe *would still have* contracted cancer if he had not smoked. If the answer is negative, then one concludes that Joe's smoking did indeed cause his cancer.

This definition is appealing in its simplicity and corresponds to the “but-for” test used in legal liability.^2^ It shifts the burden for assessing the causal claim entirely onto the determination of the counterfactual “If not *c*, then not *e*,” and thus promises a reductive account of actual causation (and potentially general-level causation too). There are, however, numerous difficulties with the standard counterfactual account (Collins et al., 2004; Moore, 2009). We will focus on the problem of over-determination, vividly illustrated by our earlier example of the shooting of the barrister in the siege. Recall that three marksmen each shot the victim, and the coroner judged that each of these shots, by itself, was sufficient to kill the victim. The counterfactual test seems to give the wrong answer in such cases. It fails to attribute causality to any of the marksmen's shots, because for any particular shot, the victim would still have died even if that shot had not been fired, as the other shots would have killed him.

There have been various attempts to solve this problem, but it is doubtful that the simple counterfactual account has the resources to deal with such cases, or a whole class of related problems (pre-emption, double prevention, trumping, see Collins et al., 2004; Moore, 2009). One attempt is to use a finer-grained description of the outcome, such that the counterfactual “if not *c*, then not *e*” comes out true. For example, if marksman A had not shot, then the victim would not have died in exactly the way he did. However, this defense is problematic. Often the outcome of interest is insensitive to small variations in its exact specification. This is particularly clear in the siege shooting. The exact details of the shooting (e.g., its precise timing) do not alter the coarser-grained outcome that the victim died, and this is the critical variable in deciding causal responsibility in this situation (cf. Woodward, 2006; Lewis, 2000).

#### 1.2.3. Structural model account of causation

The structural model framework has led to several related accounts of actual causation (Halpern & Pearl, 2005; Hitchcock, 2001, 2007a,b; Pearl, /2009^2000^; Woodward, 2003). We will focus on the account proposed by Halpern and Pearl (2005), although the key points are similar across the different versions. At the heart of the structural model approach is the idea that causality can be represented in terms of functional relations between variables.^3^ Thus, the causal relations between a set of variables is represented by a set of structural equations. These structural equations express information about the effects of potential interventions. To say that A causes B (represented by the equation B = A, or the directed graph A→B) is to claim that there are a set of possible interventions on A (forced changes in the value of that variable) that change the value of B (or the probability distribution over B's values).^4^ The functional relations between sets of variables are supposed to represent autonomous mechanisms in the world.

A causal model is a set of variables together with a corresponding set of functional relations. By necessity a causal model of any real-world setup will leave out many details. Indeed, the choice of variables will often depend on the modeler's perspective and goals, and thus have a strong degree of relativity. However, once the variables are selected, the structural equations that correctly relate these variables are determined by the world not the modeler (see Halpern & Hitchcock, 2010). In this sense, a causal model aims to represent the causal processes and mechanisms operating in the world.

To illustrate, we will construct a small-scale causal model of the siege example (Pearl, /2009^2000^, models a fictional case that is very similar). Our model only uses six binary variables; more complicated models are possible, but the simplified model captures the key variables and causal relations. The variables in the model are as follows: BA represents the proposition that the barrister aims his shotgun at the police; PO represents the proposition that the police command issues an order to be prepared to shoot; MS1, MS2, MS3, represent the propositions that marksman 1, marksman 2, and marksman 3, respectively, each shoot at the barrister; BD represents the proposition that the barrister dies. Each variable expresses a proposition that can be either true (value = 1) or false (value = 0).

**Fig. 1:**
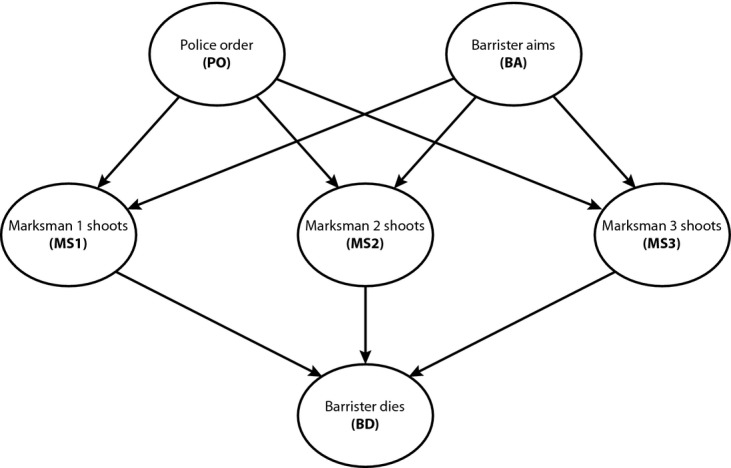
Simple causal model of siege example.

The structure of the causal model is shown by the causal graph in Fig. 1. The directed arrows from BA to MS1, MS2, and MS3 represent the claim that the barrister's aiming his gun at the police is a potential cause of each of the marksmen firing. The directed arrows from PO to MS1, MS2, and MS3 represent the claim that the police order is a causal pre-condition for the marksmen to shoot. The directed arrows from MS1, MS2, and MS3 to BD represent that each of the shots are potential causes of BD. The graph does not show the combination functions for variables that have multiple parent causes. These are specified by the form of the structural equations.

The structural equations linking the variables in the causal model are as follows:


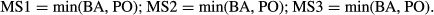


These three equations represent the assumptions that, for each of the marksmen, if the police command issue an order to prepare to shoot (PO = 1) *and* the barrister aims his shotgun at the police (BA = 1), then the marksman will shoot (e.g., MS1 = 1, MS2 = 1, MS3 = 1); otherwise none will shoot.

The key equation linking the marksmen's shots to BD is given in the following:


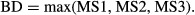


This equation represents the assumption that any one of the marksmen's shots is sufficient for the barrister to die.^5^ If one or more of the marksmen shoot (e.g., MS1 = 1), then the barrister will die (BD = 1). If none shoot, then the barrister will not die.

All of these equations are readily generalized to accommodate probabilistic rather than deterministic relations. For example, each marksman's shot might independently raise the probability of the victim dying (rather than guaranteeing it). This would correspond to the noisy-OR function in a probabilistic causal model (Pearl, /2009^2000^). For illustrative purposes, we will stick with deterministic models, but the key points carry over to the more general probabilistic context.

Before moving to questions of actual causation, it is worth highlighting some distinctive features of the causal model framework. A causal model serves as an “oracle for interventions” (Pearl, /2009^2000^) in the sense that one can use the model to predict the consequences of potential interventions. For example, we can use the current model to predict what happens if we intervene by stopping one of the marksmen from shooting (e.g., setting the variable MS1 = 0). This intervention would not alter the dependency of BD on the other two marksmen's firing (MS2 or MS3). Moreover, due to the special way in which interventions “modify” the graph structure, this intervention does not change the values of PO or BA. The ability of the causal model framework to model the consequences of interventions allows it to capture crucial components of causal reasoning and (as we shall see) is critical for the evaluation of counterfactual claims.

So far we have constructed a general-level causal model of the siege, without including specific information about what values the variables took in the actual situation. Setting up the causal model (which can involve nontrivial choices about the appropriate variables and the functional relations) is a pre-requisite for making actual causal judgments but does not determine these judgments. The next step is to consider the ascription of values to the variables in the model. A useful concept is that of a *causal world—*which is a causal model in which each variable is instantiated at a particular value (not necessarily the value it takes in the actual world). One of these causal worlds will correspond to the actual world. In the siege example, the police command did issue an order to prepare to shoot, the barrister did aim his shotgun at the police, all three marksmen fired, and the barrister died. Therefore, the actual world is represented by assigning all variables a value of one.

Note that the instantiated causal model can be used to support counterfactual reasoning (Pearl, /2009^2000^). For example, we can enquire whether the barrister would still have died if marksman1 had been distracted and failed to shoot. On the current causal model the answer is yes. The antecedent of the counterfactual involves an imagined intervention on MS1 to set its value = 0. As noted above, this does not alter the other dependencies in the model, nor does it change the fact of whether the barrister aims at the police (BA). Given that BA = 1, we can infer that the barrister would still die (BD = 1), because both the other marksmen would still have shot (MS2 = 1 and MS3 = 1).^6^

#### 1.2.4. Actual causation—counterfactual dependency under certain contingencies

We are now in a position to consider a definition of actual causation based on the structural model approach (Halpern & Pearl, 2005). We will not present the formal aspects of this definition but will summarize the key intuitions. Halpern and Pearl retain the basic counterfactual notion that one event *c* (or state of affairs) causes another event *e* if and only if there is a counterfactual dependence between the two events, such that if *c* had not occurred, then *e* would not have occurred.^7^ However, their crucial extension is that counterfactual dependence is evaluated relative to *certain contingencies* (or causal worlds), not necessarily the actual world. This allows the account to deal with cases of over-determination, pre-emption and several other classic problems for counterfactual theories. Effectively, the proposal is that a (general-level) causal model is used to establish whether a counterfactual dependence holds between a putative cause–effect pair, conditional on certain other (counterfactual) interventions on the model. In short, *c* causes *e* if and only if *c* is pivotal for *e* under certain contingencies (causal worlds), not necessarily the actual world.^8^

Of course, the plausibility of this extension depends on finding a principled way to decide which contingencies are allowable when assessing whether one event is an “actual cause” of another. Halpern and Pearl (2005) offer a complex set of conditions that aim to capture the intuition that one should only invoke contingencies “that do not interfere with active causal processes.” For example, when evaluating whether marksman1′s shot was a cause of the BD, it is legitimate to consider a possible world where the other marksmen fail to shoot (see below); but when evaluating whether the barrister's behavior (in aiming his gun at police) was a cause of his death it is not legitimate to consider a world where none of the marksmen fired. In the latter case, one is disrupting the active causal processes that are critical to the claim that the barrister's behavior caused his death. The question of which contingencies are allowable is non-trivial and is the subject of ongoing debate (Halpern & Hitchcock, 2010; Hiddleston, 2005; Hopkins & Pearl, 2003). However, for current purposes, we focus on the relatively straightforward cases of over-determination, which underpin the general framework of causal responsibility to be developed in a later section.

#### 1.2.5. Over-determination resolved

The structural account offers a novel solution to the problem of over-determination (which has plagued counterfactual theories). The basic idea is that when several causes over-determine an outcome (such that each is sufficient for the outcome), each cause qualifies as an “actual cause” of the outcome because in a causal world where the other causes are removed (by hypothetical interventions), the outcome counterfactually depends on the remaining cause. In other words, even though in the actual world the effect would still have occurred if the cause had been absent, there is a hypothetical world where the other (over-determining) causes are removed, and in that world the effect would not have occurred without the target cause.

This solution is nicely illustrated with the siege example. Consider the question of whether marksman1 is an “actual cause” of the barrister's death. In the actual world marksman1′s shot (MS1) is not pivotal for the death, because of the other two marksmen. However, consider a causal world where the other two marksmen are changed to not shooting (i.e., setting MS2 = 0, and MS3 = 0). In this world markman1′s shot *is* pivotal to the barrister's death (if the marksman1 had not fired, the barrister would not have died). The same argument applies to both of the other marksmen, so all three marksmen are ruled in as causes of the barrister's death (which conforms to our intuitive judgments about the case).

The proposed account of actual causation, and its resolution of over-determination cases, resonates with the judgments made by the experts in the siege case. Consider the post-mortem statements made with regard to the causes of the barrister's death. First, it was stated that each shot “would have been capable of causing death in their own right.” This is naturally interpreted as asserting that under causal worlds where only one marksman shoots, that individual shot would have been pivotal to the barrister's death. Second, a more subtle use of counterfactual reasoning occurs when the post-mortem states that “the likely survivability of the brain wound, heart wound or liver wound would have been very low, even if one was artificially in an intensive care unit at the time it happened.” Translated into the terminology of the structural account, this asserts that even under a contingency where the victim was placed in intensive care (and thus treated immediately), he would still have died. In this latter case, rather than simply change the values of the variables in the causal model, a new variable is introduced (immediacy of intensive care), and set at a specific value. The claim is that even under this contingency, each of the marksmen's shots would still have killed the barrister. Thus, the real-world reasoning of the coroner demonstrates the interplay between causal and counterfactuals claims, and in line with the structural model approach, suggests that causal claims are evaluated in terms of counterfactual dependence under contingency.

Note that the structural approach also sanctions the ascription of causality to the barrister's own actions. According to our simple model of the situation, if the barrister had not aimed his shotgun at the police, then the marksmen would not have fired, and the barrister would not have died. Of course, the current causal model greatly simplifies a complex situation. But it is re-assuring that it captures the key causal judgments.

#### 1.2.6. Potential shortcomings of the structural model approach

The structural model account of actual causation deals with many of the problem cases in the philosophical literature, including over-determination and pre-emption. However, it struggles with some counter examples, in particular cases where the same causal structures and variable assignments nevertheless yield different intuitions about judgments of actual cause (Hiddleston, 2005; Hopkins & Pearl, 2003). Various refinements to the structural approach have been suggested to address these shortcomings (e.g., Halpern & Hitchcock, 2010), but no consensus has been reached on these problem cases (Hall, 2007; Hitchcock, 2007a,b). More generally, the strategy of searching for necessary and sufficient conditions of actual causation, characteristic of most theories of actual causation (including Halpern & Pearl, 2005; Woodward, 2003), is open to criticism. Glymour et al. (2010) argue that this strategy has led to increasingly complex sets of conditions, as new counterexamples are encountered, but these definitions are still based on intuitions derived from only a very small subset of possible causal structures. Moreover, they show that adding causal variables to simple models can generate new problems that are not readily solved by the current definitions (and actually set alternative versions of the structural model in conflict).

This is a valid criticism of the current philosophical theories of actual causation. Although it is premature to rule out the possibility of finding a satisfactory definition of actual causation, it is possible that a unique definition will not be forthcoming (especially given the rich connotations of the notion of cause, and the many contexts in which it is used). This criticism is less pertinent to psychological models of causal judgment insofar as these models rarely consist in necessary and sufficient conditions. At most psychological models advance general principles that can be highly dependent on contextual and individual factors. However, the dangers of generalizing from a subset of causal cases are very real, as is the possibility that intuitive judgments are not stable for complex cases, and we will discuss these issues after presenting the empirical studies.

Another potential shortcoming of the structural model pointed out by Glymour et al. (2010) is that it presents a static model of actual causation, and thus does not capture the notion that a cause is often a change of state (e.g., a happening or a trigger; see also Steward, 2010). This stems from the structural model's conception of an event as a set of possible worlds rather than a transition between states. This is an important point and will be discussed in more detail in the final section. Although it does not undermine the empirical work in this article, it does suggest new lines of empirical research.

Irrespective of whether the structural model account, or successive refinements of it, can capture all the philosophical problems, we believe that the approach provides a good starting point to explore intuitive judgments of causation. It serves as a precursor for a novel account of causal responsibility, and a template for exploring psychological attributions in a principled manner.

## 2. A structural model of responsibility

Most accounts of actual causation focus on the binary question of whether something is a cause. However, in contexts where multiple agents combine to bring about an outcome (such as the overdetermination cases) it is natural to consider a more graded notion. This becomes even more pertinent when actual causal judgments are used for assignments of blame or credit. Indeed, graded judgments of responsibility are made in a broad range of social contexts—team games, business collaborations, legal sentencing, politics, medical negligence—whenever a group of agents act in concert to produce a joint outcome.

Building on the structural model of causation, Chockler and Halpern (2004, henceforth CH) propose a formal account that allows for graded degrees of responsibility and blame.^9^ They illustrate the problem with a voting example. Suppose that 11 members of a committee vote for either candidate A or B. Compare the situation where A wins the vote 11–0 with the situation where A wins 6–5. In both cases, all members who voted for A are responsible for A's victory, but a natural intuition is that the members have a higher degree of responsibility for the outcome in the 6–5 result than the 11–0 result. CH argue that this intuition is based on the fact that in the 6–5 case each member's vote was pivotal, in the sense that for each member, if they had voted the other way, then candidate A would have lost. In contrast, in the 11–0 case, no individual member's vote was pivotal in this sense. However, this is not to absolve the members of any responsibility. An important part of CH's approach is to provide a quantitative measure that distinguishes these situations, without absolving some voters of responsibility.

The key innovation in CH's account is to use the structural model account of actual cause to define a metric for assigning degrees of responsibility. Recall that *c* is an actual cause of *e* iff *e* counterfactually depends on *c* under some contingency (not necessarily the actual world). In short, *c* causes *e* iff *c* is pivotal for *e* in an appropriate causal world. Using this definition of actual cause, CH propose a definition of responsibility such that the degree to which *c*_*i*_ is responsible for *e* (out of a set of causes *c*_*1*_…*c*_*n*_), is determined by the distance between the actual world and the causal world where *c*_*i*_ is pivotal for *e*. More precisely, CH define the degree of responsibility *Resp* of *c*_*i*_ for *e* as


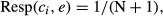


where *N* is the minimal number of changes that have to be made to the actual world in order for *e* to counterfactually depend on *c*_*i*_. Here, *Resp* is relative to a causal model and the values taken by its variables in the actual world. The fewer the number of changes needed to make *c*_*i*_ pivotal for *e*, the higher the degree of responsibility that *c*_*i*_ bears for *e*. When *c*_*i*_ is already pivotal for *e* in the actual world, then *c*_*i*_ is assigned *Resp* = 1. When *c*_*i*_ is not judged an actual cause of *e* (by the counterfactual criteria), then *c*_*i*_ is assigned *Resp* = 0.

This metric is readily illustrated with the voting example. In the situation where candidate A won the vote 11–0, none of the individual members, considered on their own, were pivotal to the outcome. Take one specific member (Fred). If Fred had voted for candidate B rather than A, A would still have won, so in the actual world Fred's vote is not pivotal for the outcome. However, suppose that we change the votes of five of the other members from A to B. That is, imagine a world where the vote is split 5–5 without counting Fred's vote. In this world, the outcome (A or B wins) depends counterfactually on Fred's vote. In other words, it takes five changes to the actual world to make Fred's vote pivotal (so *N* = 5), and thus he is assigned *Resp* = 1/(1 + 5) = 1/6. The same analysis applies to each of the 11 members who voted for A, so all of them bear responsibility = 1/6.

In the situation where A wins 6–5, each of the members is already pivotal to A's win. If any of them had voted for B, then A would have lost. Because no changes are required to the actual world (*N* = 0), each member who voted for A bears responsibility *Resp* = 1.^10^

The degree of responsibility analysis also applies to the siege example. Each marksman gets responsibility *Resp* = 1/3 for killing the barrister, because for each marksman two changes are needed to make his shot pivotal to the barristerr's death (i.e., changing the other two marksman from firing to not firing). By this analysis, the barrister's own behavior (in aiming his shotgun at the police) is assigned responsibility *Resp* = 1, because his behavior was pivotal to his death in the actual world.

One notable feature of the CH account is that it allows for more than one agent to receive full responsibility for the same outcome. In other words, it does not assume that there is a fixed sum of responsibility for an outcome that is divided among the contributing agents. This feature chimes with some philosophical discussions of moral responsibility. For example, Zimmerman (1985) argues that multiple agents can be fully responsible for the outcome of a group action, where “fully” does not mean “solely,” but rather “with no diminution.” This also accords with legal practice, where, for example, sentences for murder are not diminished merely by the number of perpetrators involved. Despite its accordance with moral intuitions and legal practice, however, it is an open empirical question whether people will assign “full” responsibility to multiple agents for the same outcome. The experimental work discussed in this article suggests that people do so, and do not restrict themselves to dividing a fixed sum of responsibility among multiple agents.

Critical to the CH account is the ability to quantify the number of changes that move one from the actual world to an appropriate hypothetical world. In the two examples above it is relatively straightforward to apply, because the variables are pre-defined and each change corresponds to a change in value of a binary propositional variable. But in some situations it might be less clear exactly what constitutes a change, and thus how changes are to be counted. A lot will depend on how the initial causal structural model is constructed. It might be argued that this is again a feature rather than a bug in the structural approach: It acknowledges that the choice of causal model and variables is an intrinsic part of any dispute about causal responsibility. This is an important issue for future work, because the proposed analysis of responsibility hinges upon the notion of changes to a causal world. We discuss the issue of the unit of change in a later section.

The definition of degree of responsibility given by CH is not yet an account of blame (or credit). It does not take into account the intentions of the agents, their knowledge or foresight about the consequences of their actions, or the justifiability of their reasons for acting (e.g., the marksmen were acting in self-defense). Such factors are crucial determinants of judgments of blame in both theory (Heider, 1958; Shaver, 1985) and practice (Gerstenberg, Lagnado, & Kareev, 2010; Hilton, McClure, & Sutton, 2010; Lagnado & Channon, 2008). To avoid any confusion on this point, we will often refer to the CH definition of responsibility as *causal* responsibility. In addition to this concept, CH propose a definition of blame that takes into account the epistemic states of the causal agents. In short, they define blame in terms of the expected degree of responsibility, relative to the epistemic state of the agent. So an agent is not automatically to blame for something he is causally responsible for; it depends also on his beliefs and knowledge. The classic example of this is the doctor who administers a drug to a patient, and the patient subsequently dies from a very unusual allergic reaction. The doctor is a cause of the patient's death, but in this case not to blame, because the outcome was completely unexpected by the doctor. (In legal cases, the situation is slightly more complex, because the critical question is whether the doctor could have “reasonably” foreseen the adverse consequence.)

Empirical evidence shows that people's blame judgments are sensitive to the epistemic states of the agents (Lagnado & Channon, 2008). The CH model can explain some of these data, but it does not account for the role of an agent's intentions. This means that the CH model is not a complete model of blame attributions, although it does capture some important features. Future developments could include explicit causal modeling of the internal mental states of the agents, such as their beliefs, desires, intentions, and reasons.

In what follows we concentrate on contexts in which multiple agents contribute to the same outcome, where the agents are members of the same group or team, and share the same beliefs, knowledge, and intentions. More complex situations can be explored at a later stage, but for now we restrict ourselves to testing the bare bones of the structural approach. Moreover, at this point we look mainly at whether people's intuitive attributions *conform* to the formal model, without close scrutiny of the psychological processes that underpin these judgments.

### 2.1. Intuitive judgments of responsibility

The structural model approach presents a formal framework for assigning causation and responsibility that aims to match our reflective (considered) judgments. It provides a principled method for attributing responsibility and can deal with contexts in which multiple agents contribute to the same outcome. How well does this approach apply to people's intuitive attributions, especially in group contexts, where over-determination is rife? As noted, this is a critical question in psychology, where responsibility attributions are a prevalent part of social interactions, in diverse areas such as sport, law, business, politics, and everyday gossip. Before presenting a novel experiment that addresses this question, we review some previous empirical tests of the structural model approach (and the CH model in particular).

### 2.2. Sensitivity to causal structure

A central claim of the structural approach is that responsibility judgments are sensitive to causal structure, both in terms of the network of causal relations between the relevant actions and events (the causal graph), and the causal functions that dictate how multiple causes combine to yield an outcome (given by the structural equations). The latter is particularly relevant in group situations where the individual contributions of each member are combined to determine the overall group result. Three common functions are summation, conjunction, and disjunction (cf. Steiner, 1972). In the *summative* case, each individual member contributes to the group result, and their contribution is proportional to their own individual performance. For example, in a tug-of-war, each member of the team contributes to the overall team performance, and this contribution is proportional to their individual “pulling power.” In the *conjunctive* case, each member also contributes to the group outcome, but here each member must satisfy a specific threshold or criterion. Thus, when individual performance is measured by a binary variable (e.g., pass or fail), a conjunctive case requires that every member pass for the team to win. For example, consider a human triangle in an acrobatic display. If any one of the acrobats fails in his or her positioning, the team will fail overall. In the *disjunctive* case, the performance of a single individual is sufficient for the team outcome. Thus, in the binary variable context, so long as one team member passes the team wins. A real-world example is a team quiz, where the team wins a point as long as one member of the team gets the right answer.^11^

Intuitively, each of these combination functions can imply a different pattern of responsibility attributions. This is because the relation between an individual member's performance and those of his team members varies across these functions. The structural model makes explicit predictions about how responsibility attributions should vary depending on the functional relations between team members. According to the structural model the degree of responsibility attributed to a team member for the team's result depends on that member's own performance (e.g., his success or failure in his individual task) and the performance of other members, because if the outcome is over-determined, the actual world needs to be changed to make the agent pivotal (the more changes needed, the less responsible the member is). This makes responsibility attribution sensitive to the combination function, and implies distinctive patterns in disjunctive versus conjunctive contexts.

Imagine a simple game setup, where a team has four members, each of whom can pass or fail their individual task. With a disjunctive function, only one individual pass is needed for the team to win the game. In the case where only one team member passes his task, attribution is simple: that member gets full responsibility for the win (*Resp* = 1) and the other members get nothing (*Resp* = 0). Now consider the situation where two members pass. The win is over-determined, and neither member is pivotal. According to the structural model, both these members get responsibility *Resp* = 1/2, because for either of them it would take one change to make them pivotal to the team win (i.e., by changing the other member from pass to fail). A similar argument applies when three members pass, with each of these members receiving *Resp* = 1/3, and when four members pass, with each receiving *Resp* = 1/4. More generally, for each member who passes their individual task, their responsibility for the team win increases as the number of other successful members decreases. In comparison, consider the case where none of the members succeed and therefore the team loses. All members have full responsibility (*Resp* = 1) for the team loss, because each is pivotal (in the actual world) for the result. If any of them had succeeded in their task, the team would have won.

In contrast, consider a conjunctive function, where the team only wins if all four members pass their individual tasks. When the team wins, each member is pivotal: If any one of them changes from pass to fail, then the team loses. Thus, each member is fully responsible for the win. However, things are different when the team loses. Consider the situation where all members fail. In this case, each member gets *Resp* = 1/4 for the team loss, because three changes need to be made to make a member pivotal. Now consider the situation where two members succeed. The two members who fail now receive *Resp* = 1/2 for the loss. More generally, as the number of members who succeed increases, the responsibility of the member who fails increases (until the limit where that member is the only one who fails, and is assigned full responsibility for the loss).

To summarize, the structural model implies the following patterns: for disjunctive games, the degree of responsibility assigned to a successful member (for a team win) *decreases* with additional members who succeed, whereas all members get full responsibility for a team loss; for conjunctive games, the degree of responsibility assigned to an unsuccessful member (for a team loss) *increases* with additional members who succeed, whereas all members get full responsibility for a team win.

### 2.3. The triangle game

Gerstenberg and Lagnado (2010) constructed a novel experimental paradigm to explore some of the predictions of the structural model. Participants played “the triangle game” in teams of four players including the participant and three hypothetical players and then attributed responsibility to each player (including themselves) for the team's win or loss using a sliding scale from 0 to 10. In the triangle game, each player is asked to estimate the number of triangles in a complex figure under a tight time constraint. The deviations of the individual answers from the correct answer determined the team outcome according to a rule that differed in three between-subjects conditions. In the *Sum* condition, the team won if the sum of the deviations of each player was six or less. In the *Max* condition, the team won if all players deviated by 2 or less. Finally, in the *Min* condition, the team won if at least one of the players answered correctly. Note that in the *Max* condition, the function that determines the team outcome is conjunctive for a win but disjunctive for a loss, whereas in the *Min* condition, the function is disjunctive for a win, but conjunctive for a loss.

Gerstenberg and Lagnado (2010) found that the structural model (Chockler & Halpern, 2004), which assigns responsibility on the basis of the minimal number of individual outcomes that have to be altered to make a player under consideration pivotal, explains responsibility attributions better than a simple counterfactual model. However, responsibility attributions were also sensitive to the deviation of a player's answer from the correct answer. Larger deviations were associated with more blame for losses and less credit for wins, even when they did not affect the team outcome. For example, a deviation of 3 and a deviation of 4 in the *Max* condition both fail to pass the maximal criterion of 2 deviations, but the former attracted less blame for a team loss compared to the latter. This finding will be addressed in a later section of this article.

**Fig. 2:**
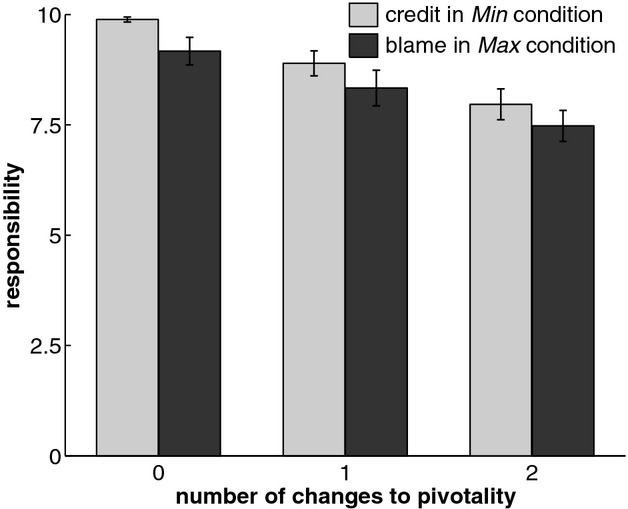
Mean responsibility attributions in situations of over-determination in the triangle game of Gerstenberg and Lagnado (2010) separately for credit in the *Min* condition and blame in *Max* condition. *Note*: Error bars indicate ±1 SE.

Here, we complement the analysis in Gerstenberg and Lagnado (2010) by focusing on over-determination situations, as they occur in the *Min* and *Max* conditions. Fig. 2 shows the credit attributions in the *Min* condition and the blame attributions in the *Max* condition as a function of the minimal number of changes to outcomes of other players required to make a player pivotal. As predicted by the structural model, credit in the *Max* condition and blame in the *Min* condition to an individual player decreased with the number of changes that would have been required to make the player pivotal.

In summary, the data provided support for the structural model, although people's judgments were sensitive to the degree to which player's estimates deviated from the correct answer, irrespective of whether a threshold criterion was reached.

### 2.4. Complements and substitutes

The data from the triangle game show that responsibility decreases with the number of players who share it in situations of over-determination. However, this pattern can also be explained by a simple diffusion of responsibility model (cf. Latané & Darley, 1968), in which a fixed amount of responsibility is shared among all players that succeed (or fail) in their individual tasks. In contrast, a central feature of the structural model is that the responsibility assigned to an individual team member depends in a nontrivial way on the performance of that player's teammates, and the causal function that combines each member's performance into the team outcome. Zultan et al., (2012) introduced two asymmetric team games to allow for a clean comparison of the effect of one group member's outcome on the responsibility attributed to another. In their experiment, the individual task consisted of the *dot-clicking game*, in which a dot on the computer screen is repositioned each time the player clicks on it. The goal is to click on the dot a certain number of times within a given duration. Participants practiced playing the game themselves, but in the judgment phase of the experiment they took the role of external observers and attributed blame to individual players whose team lost the challenge.

**Fig. 3:**
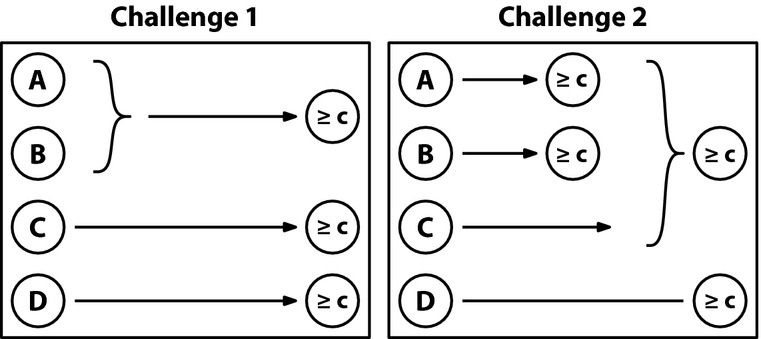
Diagrams of two different team challenges used in Zultan, Gerstenberg, and Lagnado (2012) which differed in terms of which players have to reach a performance criterion ≥ *c* in order for the team to win the challenge. *Note*: Curly braces indicate that contributions combine in a disjunctive fashion.

The two team challenges are presented in Fig. 3. In Challenge 1, both players C and D as well as at least one out of A and B have to succeed in order for the team to win the challenge. In Challenge 2, D has to succeed and either C or both A and B. In Challenge 1, player A is a substitute of player B, but a complement of player C, whereas in Challenge 2 the opposite holds. Players are *substitutes* if their individual contributions combine in a disjunctive fashion and *complements* if their contributions combine conjunctively. Compared to a baseline in which all players failed, the structural model predicts that A's blame will increase if his complement succeeded (e.g., C in Challenge 1 and B in Challenge 2). If A's complement succeeded, one less change is needed to make A pivotal for the loss. In contrast, the model predicts that A's blame will *decrease* (compared to the baseline) when his substitute succeeded (e.g., B in Challenge 1 and C in Challenge 2). In a situation in which A's substitute succeeded, one more change is needed (namely changing the substitute to having failed) to make A pivotal compared to the baseline.

Zultan et al. (2012) varied the number of participants who failed in their task: either all four players, all-but-B or all-but-C.^12^ A simple diffusion model predicts that the blame assigned to player A increases when the number of group members who failed decreases (as there are fewer people now who share the blame). In contrast, the actual blame attributed to player A was always lower when his substitute peer succeeded than when his complementary peer succeeded. Thus, player A received more blame in Challenge 1 when player C succeeded (his complement) than when player B succeeded (his substitute). In Challenge 2, in which the relationships of complementarity and substitution were reversed, A was blamed more when B succeeded than when C succeeded. While these predictions follow naturally from the structural model, a simple diffusion of responsibility cannot explain the differences to A's blame since the same number of players failed in each situation.

The results also showed an effect that was not predicted by the structural model: In Challenge 1, participants blamed players C and D more than A and B in a situation in which all players failed. However, the minimal number of changes to make, for example, A or C pivotal is the same. Two changes are required to make A pivotal, namely changing C and D. Similarly, a minimum of two changes are needed to make C pivotal, for example, changing D and A. However, note that there is an important asymmetry between A and C. Whereas there is only one way to make A pivotal (through changing C and D), there are multiple ways to make C pivotal (through, e.g., changing D and A or D and B). Zultan et al. (2012) extended the structural model to allow for different ways in which a player can counterfactually become pivotal. This “multiple-paths” model provides a formal way to attribute higher responsibility to individuals who are pivotal in more than one hypothetical world, taking into account both the number of such hypothetical worlds and their “distance” from the actual world. The extended model correctly predicts that player C incurs more blame than player A in the described situation.

## 3. Pivotality and criticality

The results from Gerstenberg and Lagnado (2010) and Zultan et al. (2012) confirm that the structural approach to responsibility successfully explains attributions to players who, in retrospect, would not have altered the team outcome. It achieves this by using a graded notion of responsibility determined by the number of changes needed to render a player pivotal for the outcome. The approach does not allow, however, for graded attributions of responsibility with regard to players who are already pivotal. Nonetheless, certain cases generate a strong intuition that pivotal individuals in different situations are not regarded as equally responsible. Consider the classic bystander effect (Latané & Darley, 1968): A victim is attacked by an offender and is in need of help. Several observers are in a position to potentially intervene. As is well known, people have a reduced sense of responsibility when there are others who could also help compared to a situation in which there is no one else. However, note that the structural model does not predict this effect. The structure of the situation is disjunctive (assuming that one person would be enough to fend off the offender) and thus each person is pivotal for the negative outcome, irrespective of the number of people present.

This intuition can be accommodated within the structural framework by incorporating the concept of prospective responsibility: the extent to which a person is perceived to be critical for an outcome *before* it has occurred. Accordingly, the reason why a single person will be held more responsible for not helping a stranger than each individual in a group of people, is that the single person could have prevented the outcome (he was pivotal) *and* he knew that the nature of the outcome only depended on him (he was more critical). Thus, *retrospective* responsibility is not only influenced by whether a person was pivotal *after* the fact but also by how critical a person was perceived to be *prior* to the outcome.

We show that incorporating the notion of criticality explains additional deviations from the predictions of the structural model and we present a new experiment to test the respective roles of criticality and (distance from) pivotality in intuitive judgments of responsibility.

The new framework subsumes the “multiple-paths to pivotality” model introduced and tested in Zultan et al. (2012). As we shall see, prospective responsibility reflects different possible ways in which one can be pivotal, and as such is closely related to the multiple-paths model. In particular, the new framework predicts the patterns observed in Zultan et al.'s experiments. However, the new proposal goes beyond the multiple-paths model insofar as it explicitly incorporates people's judgments of criticality. Thus, it predicts that retrospective responsibility assignments can differ even when agents are equally pivotal for the outcome.

### 3.1. Models of criticality

Let us summarize the key intuitions with respect to criticality: An individual's perceived criticality diminishes with an increased number of people in disjunctive structures because the actions of each individual in the group are alone sufficient to bring about the outcome. However, an individual's criticality is not expected to decrease with an increased group size for conjunctive tasks. Regardless of the number of conjuncts, each individual's action is still necessary for the team outcome. To illustrate, imagine that a victim is attacked by *three* offenders and three observers are present, each of whom would need to intervene in order to help the victim. Here, the intuition is that each of the observers is highly critical for preventing the outcome (and more critical than in a situation in which only one offender was present but there were still three observers).

So far we have relied on intuitive perceptions of criticality and have not given a formal model of criticality. We now consider different ways in which the intuitions discussed above can be formalized and generalized. These will allow us to define a criticality-pivotality framework to be tested in a new experiment. We consider two possible models of criticality, which we shall refer to as the *expected pivotality model* and the *heuristic model*. These models are intended as an example for how criticality can be formalized and incorporated into a model of retrospective responsibility. Other approaches to criticality may be just as valid as the ones we discuss here. We will mention one such alternative approach when discussing the heuristic model.

The *expected pivotality model* is based on Rapoport (1987), who provides a definition of the criticality of a person's contribution in the step-level public goods game. In this game, each person in a group is endowed with an initial amount of money, let's say $5. Each person then indicates whether he or she wants to contribute money to the public good. If a sufficient number of people provide their endowment and the provision point is met, the public good is provided (e.g., each person gets an additional $10). The public good is available to all in the group, no matter whether they contributed their endowment. According to Rapoport's (1987) definition, a player's criticality is given by the probability that his or her contribution will make a difference to the outcome. In the step-level public goods game, this is the probability that the number of contributors among the other players is exactly one less than the number required to provide the public good, given the prior probabilities regarding contributions in the group. In our more general setup, this is equivalent to the ex ante probability that a player's contribution will be pivotal.

Does this definition of criticality as “expected pivotality” capture our intuitions with regard to the examples described above? It correctly predicts that an individual's criticality reduces with an increased group size in disjunctive situations. However, contrary to intuition, the model also predicts that criticality will be similarly reduced in conjunctive tasks. To see why, note that in disjunctive tasks, a player is pivotal if *none* of the other players succeed. In comparison, in conjunctive tasks, a player is pivotal if *all* of the other players succeed. Assuming that a player is maximally uncertain about whether or not the others will succeed, the probability of him being pivotal is the same in the disjunctive and conjunctive tasks and reduces with the size of the group.

**Fig. 4:**
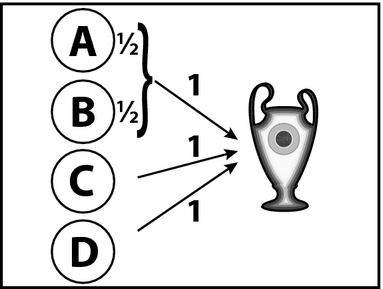
Causal structure of asymmetric example with the predictions of the heuristic criticality model.

The second model of criticality we consider is a simple heuristic model. This model assigns full criticality to players whose success is necessary for the team outcome and divides the criticality equally between players who share a task in a disjunctive fashion. This is best illustrated via the asymmetric task structure used in Zultan et al. (2012) and reproduced in Fig. 4. In this challenge, both *C* and *D* have to succeed in their individual tasks in addition to at least one player out of *A* and *B*. The heuristic model assigns full criticality to *C* and *D*, whereas *A* and *B* only receive a criticality of 0.5 each, because their contributions combine in a disjunctive manner.

Another way to formalize the importance of a player whose success is necessary for the team to win is to model criticality as the relative decrease in the probability of the team winning when the player fails. For example, this notion of criticality can be defined as 
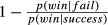
 According to this definition, a player's criticality varies from zero (when he has no effect on the team outcome) to one (when he is necessary for the team to win). The predictions of this model for the deterministic situations to be explored in the next section are virtually identical to the predictions of the heuristic model. We will, therefore, not discuss it separately in the following, but present it here as an illustration for how models of criticality can be extended to encompass non-deterministic situations.^13^

### 3.2. Testing the criticality-pivotality model of retrospective responsibility

We designed a new experiment to investigate the influence of both criticality and pivotality on participants' responsibility attributions. We hypothesized that participants' responsibility attributions would not only be affected by how close a player's contribution was to being pivotal but also by how critical this player's contribution was perceived for the group outcome. Formally,


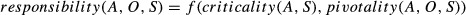
where *criticality(A,S)* denotes the criticality of player *A* in situation *S* and *pivotality(A, O, S)* denotes *A*'s pivotality for the outcome *O* in situation *S*. In the following experimental study, we illustrate how the general framework can be applied using the different models of criticality and pivotality discussed above.

**Fig. 5:**
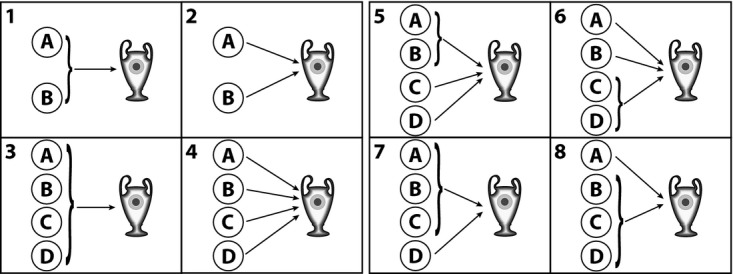
Team challenges used to investigate the effects of criticality and pivotality on responsibility attributions to player A.

In the experiment, 40 participants (25 female) aged 18–60 (*M* = 33.86, *SD* = 11.76) were recruited online via Amazon Mechanical Turk.^14^ They evaluated the performance of contestants in a game show in which players played the dot-clicking game (for details see above discussion of Zultan et al., 2012) and were randomly assigned to team challenges that differed in terms of group size and structure (see Fig. 5).

In the first part of the experiment, participants viewed four different challenges on the same screen and were asked to answer the following question: “How critical is Player A for the team's outcome in each challenge?” Participants made their judgments on separate sliders, which were positioned under the four different challenges. The endpoints of the sliders were labeled “not at all” and “very much.”

**Fig. 6:**
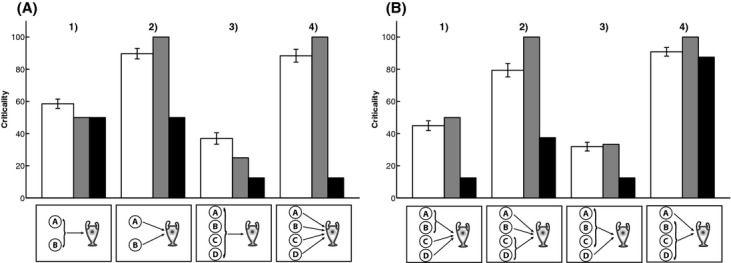
Mean criticality judgments for player *A* (white bars) and model predictions by the *heuristic model* (gray bars) and the *expected pivotality model* (black bars) for two different sets of challenges. (A) First set of challenges; (B) second set of challenges. Error bars indicate ±1 SE.

Participants' criticality judgments are shown in Fig. 6. The *heuristic model* (*r* = .97, *RMSE* = 11.15) predicted participants' criticality judgments very well and better than the *expected pivotality model* (*r* = .62, *RMSE* = 37.42). Generally, player *A* was rated highly critical when his individual success was necessary for the team win (cf. challenges 2 and 4 in Fig. 6A and B). When *A* formed part of a disjunctive (sub-)group, criticality reduced with the number of people in the group (cf. challenges 1 and 3 in Fig. 6A and B).

**Fig. 7:**
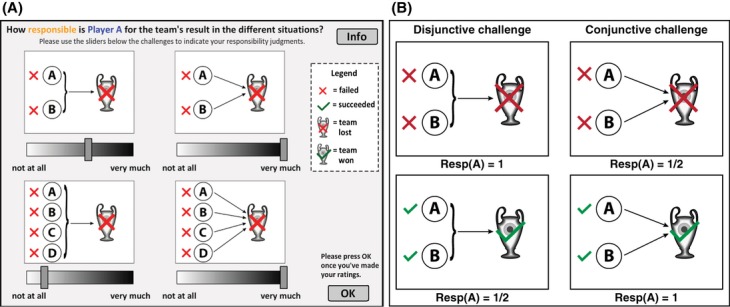
(A) Screenshot of the experiment and (B) predictions of A's responsibility by the *structural model* for disjunctive versus conjunctive tasks in which two players failed (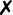) or succeeded (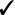).

In the second part of the experiment, participants saw the results of four different group challenges simultaneously on the screen (see Fig. 7A) and answered the following question: “How responsible is Player *A* for the team's result in the different situations?” Participants indicated their responses on separate sliders whose endpoints were labeled “not at all” and “very much.”

As a reminder, Fig. 7B shows the predictions of the Structural Model for four simple situations. As outlined above, the model predicts that in a situation in which two players failed their tasks, A's responsibility is 1 in a disjunctive challenge and 1/2 in a conjunctive challenge. Conversely, the model predicts that when both players succeeded, A's responsibility is 1/2 for disjunction and 1 for conjunction. However, remember that people perceive A to be more critical for the team's outcome in the conjunctive versus the disjunctive challenge.

**Fig. 8:**
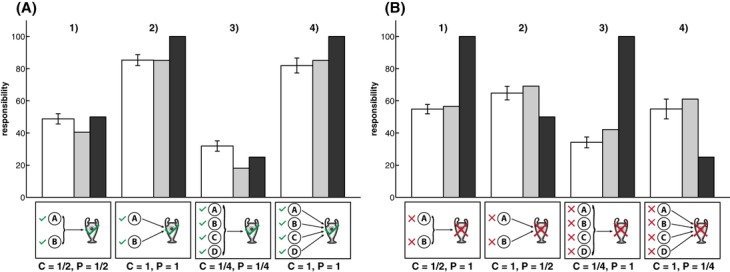
Mean responsibility ratings (white bars) for two sets of challenges with the predictions of the *critical-pivotality model* (gray bars) and the *structural model* (black bars). *Note*: C = Criticality; P = Pivotality. (A) All players succeeded; (B) All players failed. Error bars indicate ±1 SE.

In the experiment, participants saw nine sets of challenges, which were chosen to manipulate pivotality while keeping criticality constant and *vice versa*.^15^ Fig. 8 shows participants' responsibility judgments for two sets of challenges that incorporate the four situations shown in Fig. 7B. The *structural model* predicts participants' responsibility attributions well for the situations shown in Fig. 8A but not for those in Fig. 8B. Note, however, that in Fig. 8A, criticality (as predicted by the *heuristic model* which explains participants' criticality attributions best) and pivotality are perfectly confounded, whereas in Fig. 8B, criticality and pivotality are negatively correlated. In this latter set of challenges, participants attributed more responsibility when A failed in conjunctive tasks versus disjunctive tasks, in sharp contrast to the predictions of the *structural model*. For example, although *n* = 3 changes would be necessary to make A pivotal when four players failed in the conjunctive challenge (Situation 4), A receives more responsibility than in the situation in which four players failed in the disjunctive challenge (Situation 3), although A is pivotal in this situation. Note that the “multiple-paths model” (Zultan et al., 2012) also predicts that A will be held more responsible in Situation 3 than in Situation 4. The multiple-paths model assigns full responsibility to pivotal players (Situation 4) and, given that there is only one way to make A pivotal in Situation 3, makes the same prediction as the structural model in this case. However, our novel criticality-pivotality framework can account for participants' attributions by assuming that participants' weighed criticality more heavily than pivotality in this set of patterns. The fact that non-pivotal players are sometimes attributed more responsibility than pivotal players highlights the importance of criticality considerations for people's attributions.

**Fig. 9:**
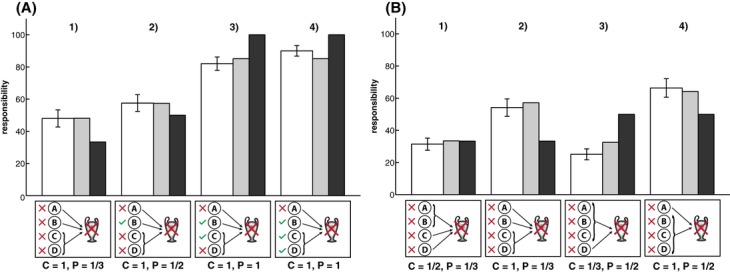
Mean responsibility ratings (white bars) for two more sets of challenges with the predictions of the *critical-pivotality model* (gray bars) and the *structural model* (black bars). (A) Criticality constant, pivotality varied; (B) pivotality constant, criticality varied. *Note*: C = Criticality; P = Pivotality. Error bars indicate ±1 SE.

From this pattern of results, one might infer that pivotality is not essential for attributions of responsibility, because participants' attributions closely follow the predictions of the criticality heuristic. However, our design also included situations in which A's criticality was held constant but his pivotality varied. If pivotality is not important, then A's responsibility should be the same in these situations. Consider the set of challenges shown in Fig. 9A. Because the structure of the challenge did not vary between situations, A's criticality is equal in all four situations. However, participants attributed more responsibility to A when he was pivotal (Situations 3 and 4) compared to when he was not pivotal (Situations 1 and 2), *t*(39) = 6.63, *p* < .001, *r* = .53. In fact, the pattern of attributions here exactly follow the predictions of the *structural model*: A's responsibility is lowest in Situation 1 (*M* = 48, *SD* = 33.75) where *n = 2* changes are required to make him pivotal. A's responsibility increases slightly in Situation 2 (*M* = 57.55, *SD* = 32.92) as *N* is reduced to 1. Responsibility further increases significantly in Situation 3 (*M* = 82.05, *SD* = 26.34) in which A is pivotal and only minimally in Situation 4 (*M* = 90.03, *SD* = 20.82).

The set of challenges shown in Fig. 9B includes two pairs of situations in which A's pivotality was the same but his criticality differed. In Situations 1 and 2, *N = 2* is required to make A pivotal. However, A is more critical in Situation 2 than in Situation 1. Participants judged *A* significantly more responsible for the team's loss in Situation 2 (*M* = 54.18, *SD* = 34.29) than in Situation 1 (*M* = 31.43, *SD* = 23.98), *t*(39) = 6.01, *p* < .001, *r* = .48. In Situations 3 and 4 A's pivotality is 1/2; however, A is more critical in Situation 4 than in Situation 3. Participants judged *A* significantly more responsible in Situation 4 (*M* = 66.35, *SD* = 36.41) than in Situation 3 (*M* = 25.13, *SD* = 21.38), *t*(39) = 9.09, *p* < .001, *r* = .68.

Taken together, the four sets of challenges discussed so far establish that responsibility attributions to individuals within a group are affected both by how critical the person is perceived to be and by how close he was to being pivotal. When criticality is held constant (cf. Fig. 9A), responsibility increases with pivotality. When pivotality is held constant (cf. Fig. 9B), responsibility increases with criticality. Thus, neither criticality nor pivotality alone is sufficient to explain responsibility attributions. This conclusion holds regardless of the particular models of criticality and pivotality used. In the following section, we proceed to illustrate how specific models can be employed to derive quantitative predictions about how responsible an individual will be judged in different situations.

### 3.3. Testing the criticality-pivotality framework

We first test how well simple models that do not combine criticality and pivotality can explain participants' responsibility attributions. For models for criticality, we consider the *expected pivotality model* and *heuristic* model described above. For models of pivotality, we consider a *simple counterfactual model*, which only assigns responsibility to pivotal players and the *structural model*, which assigns responsibility as a function of the distance to pivotality. The diagonal in Table 1 shows how well these simple models predict participants' *responsibility attributions* in the experiment. In general, the two *pivotality models* predict participants' attributions better than the two *criticality models*. More precisely, the *structural model* predicts participants' attributions best followed by the *simple counterfactual model*, the *heuristic model*, and the *expected pivotality* model.

**Table 1:** Correlations (with *RMSE* in parentheses) of *criticality models* and *pivotality models* with participants' responsibility attributions

	Criticality		Pivotality	
	Expected Pivotality	Heuristic	Simple Counterfactual	Structural
Expected pivotality	0.59 (24.44)	–	–	–
Heuristic	0.71 (19.57)	0.67 (22.41)	–	–
Simple counterfactual	0.83 (16.12)	0.89 (13.92)	0.74 (36.14)	–
Structural	0.84 (13.66)	0.90 (10.81)	0.74 (16.55)	0.77 (19.59)

*Note*. Cells on the diagonal show predictions of simple models; cells off the diagonal show predictions of combined models.

We also considered models that predicted responsibility as a weighted function of criticality and pivotality. Thus,


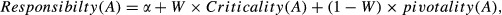


where *α* is a global intercept and *w* is a weighting parameter whose range is constrained from 0 to 1.^16^ The cells off the main diagonal in Table 1 show the model fits for these weighted models. Overall, a model that uses the *heuristic criticality model* and the *structural pivotality model* explains participants' attributions best. However, a model that replaces the *structural pivotality model* with a *counterfactual pivotality model* also explains the data well. This is not surprising, given that the predictions of the *structural* and *counterfactual* model are strongly correlated (*r* = .97) for the set of situations we employed. As a check, we also included weighted models that combined two models from the same family (i.e., two *criticality models* or two *pivotality models*). These models perform much worse than those that combine one model from the criticality family with one model from the pivotality family.

**Fig. 10:**
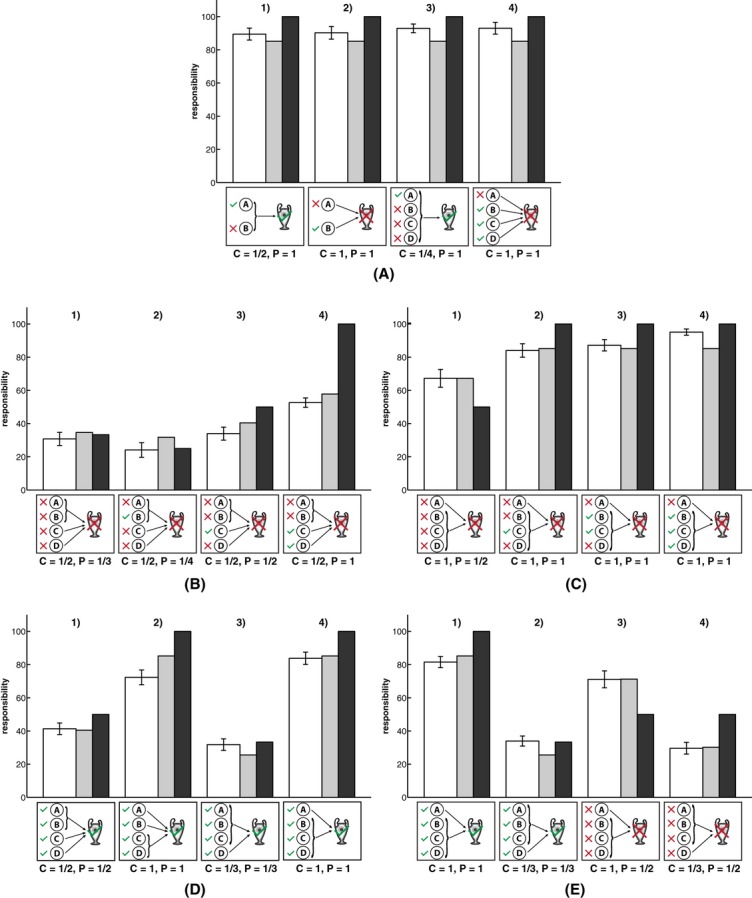
Mean responsibility ratings (white bars) for the remaining five sets of challenges with the predictions of the *critical-pivotality model* (gray bars) and the *structural model* (black bars). *Note*: C = Criticality; P = Pivotality. Error bars indicate ±1 SE.

Although the overall correlations of several combinations of models are high, the specific sets of challenges we have discussed so far support a combination of the *criticality heuristic* with the *structural model of pivotality*. For example, the pattern of attributions in Fig. 9A in which criticality was held constant is captured by the *structural model of pivotality* but not the *simple counterfactual model*. The simple counterfactual model cannot predict that A is held responsible, even in situations in which he was not pivotal. Similarly, participants' judgments for the situation shown in Fig. 8B cannot be explained if the *expected pivotality model* is used as a model of criticality, but it follows naturally when the *heuristic model* is used. The *expected pivotality model* cannot predict this pattern since it assigns equal criticality to players in disjunctive and conjunctive structures as discussed above. Fig. 10 shows the five remaining sets of challenges. We will not discuss these patterns of results in any detail; however, the patterns of attributions within each given set of challenges is consistent with the *critical-pivotality model* that combines the heuristic with the structural model. This is seen by comparing the actual judgments (white bars) with the *critical-pivotality model* (gray bars). In contrast, for each of the other possible combinations of models, there are qualitative patterns that cannot be explained.

Thus, the overall results of the experiment show that participants' responsibility are very well explained by assuming that they take into account both: (a) how critical a person's contribution was for the group outcome, and (b) whether or not a person's contribution would have made a difference to the outcome (and if not, how close it was to making a difference). As noted above, we have applied a heuristic model of criticality, but a more sophisticated probabilistic model yields equivalent results. It also has the benefit of generalizing to more complex structures. Our framework assumes a simple linear combination of both criticality and pivotality. Future research is required to develop a refined model of criticality and combine both criticality and pivotality into an integrated model of responsibility attribution.

## 4. General discussion

This article had two major aims: (a) to introduce the structural model approach as a framework for exploring attributions of causal responsibility; and (b) to present and empirically test a new model of intuitive judgments of responsibility that combines both pivotality and criticality. Before summarizing the new model in the conclusions section, we will consider the generalizability of the findings and some future extensions of the model and new directions for research.

### 4.1. Generalizability of responsibility model

For the simple cases that we have investigated so far, people appear to make stable and systematic attributions that can be captured by a few well-motivated principles (e.g., pivotality and criticality), with causal structure playing an important role. An important line for future research is to investigate whether these principles scale-up to more complex cases (or when additional factors, such as intentions and foresight are introduced). For example, what happens when there are more complicated causal structures, with dependencies between agents, such as hierarchical structures (e.g., a management structure and their employees; a coach and the team players)? What about contexts that involve sequential rather than simultaneous subtasks (Gerstenberg & Lagnado, 2012) or where the agents differ in their capabilities (Gerstenberg, Ejova, & Lagnado, 2011)? As noted in the introduction to this article, these more complex contexts raise many intriguing questions. It is possible that the current principles (e.g., of pivotality and criticality) might need to be revised or new principles introduced. However, the causal model framework, understood as a flexible and guiding framework, helps us formulate and examine many of these questions empirically. This connects with some of the criticisms leveled by Glymour et al. (2010) at the philosophical accounts of actual causation based on structural models. As noted above, we agree with Glymour et al. that the search for a set of necessary and sufficient conditions to define actual causation is not the most appropriate strategy. This holds for psychological theorizing even more than philosophical theorizing. Instead, the development and testing of core principles, with room for contextual and individual differences, seems more appropriate and is consistent with theorizing in other areas of psychology.

This connects with a related question about the possibility of individual differences in people's attributions. Although people's judgments appear robust for the simple cases, it is likely that there might be individual differences in people's intuitive attributions for more complex cases (especially when different principles might be in tension). Here again, a flexible overarching framework can accommodate these differences in a principled way. For example, the explicit model-dependence espoused by the structural framework provides one route to deal with individual differences (in particular, when scenarios are under-described, and thus allow people to fill in the gaps in different ways). Another route is to investigate different “parameter” settings of the model, for example, differential weighting of the exact combination of pivotality versus criticality in retrospective responsibility judgments. These questions have not been systematically explored but suggest a fruitful area for future research.

#### 4.1.1. Shared versus group responsibility

Although the experiments in this article have looked at team games, the focus has been on individual causal responsibility within a team. However, an important aspect of legal accounts of responsibility is the distinction between shared and group responsibility (Cane, 2002). The former is still based on individual responsibility: Each individual shares responsibility for an event. In contrast, the latter treats the group as an independent entity. Applied to the barrister siege example presented in the introduction, the inquest investigated the individual responsibility of each marksman for the barrister's death, but did not examine group responsibility overall. Some of the jury recommendations, however, seemed to be directed at the police as a group entity. Criminal law deals predominantly with individual or shared responsibility, although there are exceptions (e.g., finding corporations guilty of manslaughter in large-scale transport disasters). The theoretical and empirical work in this article has also concentrated on individual and shared responsibility (i.e., the distribution among individuals), but there are many interesting questions that could be asked about the notion of group responsibility, too.

#### 4.1.2. Hierarchical structures

Another restriction in the current article is the use of simple group structures, where the causes are independent (i.e., separate agents succeeding or failing in their own task). But in many group situations, the causal structure is more complex, including causal interactions between the agents and chains of command. Thus, when considering the allocation of responsibility, the hierarchal structure of the group is often crucial (e.g., boss and employees; manager and players; commanders and soldiers). For example, in the inquest into the barrister siege case, the jury did not find the individual marksmen legally responsible but directed blame at the police's overall handling of the event. The attribution of responsibility in hierarchical structures is a controversial and fascinating issue, but it has received sparse attention in the psychological literature (see Hamilton, 1978, 1986). We believe that the structural model framework is readily extended to address such hierarchical contexts (see Halpern & Hitchcock, unpublished data, for suggestions in this direction).

### 4.2. Model-dependence of responsibility judgments

On the structural model approach, responsibility judgments are explicitly model dependent. Thus, even when presented with an identical scenario people might construct different models and hence legitimately differ in their responsibility judgments. In the experiments used so far (e.g., the dot-clicking game), the causal structures were clear and unambiguous, so it is unlikely that participants constructed different causal models. However, in richer and more complex cases, people might differ in their causal models and hence also in their responsibility attributions. As noted above, this is one route by which individual differences in judgments might be explained, especially in complex contexts with multiple causal factors. To investigate this issue, future work could independently elicit peoples' causal models (and thus directly investigate the effect of model choice on responsibility attributions). More generally, the question of how people build causal models of complex situations is under-researched (see Fenton, Neil, & Lagnado, 2012, for a proposal in the legal context).

### 4.3. Unit of change

Another important component of the structural model is the number of changes needed to make an agent pivotal to the outcome. Chockler and Halpern (2004) rely on examples where the unit of change is straightforward—either single votes in the election example, or shooting versus not shooting in the marksman example. More generally, the structural model framework suggests that one clear-cut unit of change is the value of a variable. This is readily applied to the dot-clicking game explored in our studies (and numerous real-world contexts). The simplest case is when all variables are binary, so a single change involves switching the value of a single variable (e.g., changing a player from failure to success in their individual task). Things are more complicated when the variables are multi-valued, as in the triangle game. One unit of change is the point score (deviation) of an individual player; another could be a wholesale change in the player's score. The choice of unit here can make a difference to assignments of responsibility because it can entail different numbers of changes required for pivotality. For example, consider the *Max* condition in the triangle game, where each player's individual deviation from the correct answer must be less than 3. Compare two cases in which A and B are over the threshold: (a) players A and B both deviate by 3; (b) player A deviates by 3, player B deviates by 5. Is player A equally responsible in both cases? If the unit of change is at the level of player, then in both cases only one change is required to make A pivotal, namely, to switch player B so that his score is under the threshold. However, if the unit of change is deviation points, then a greater number of changes is needed in case (b) than (a), and thus player A is assigned less responsibility in case (b).

This example highlights the fact that the level of grain of the unit of change can affect how many changes are needed for pivotality, and thus the degree of responsibility assigned. It is not a criticism of the structural approach, but it does show that care needs to be taken in defining the unit of change for any given context—and suggests that this might be a significant factor in people's own intuitive judgments.

As well as considering changes to values of variables, it is possible to consider more substantial changes, such as modifications to the causal models. This might involve the introduction of new variables, or changes to the causal relations or functions between variables. An example of the former is given in the siege example, where the possibility of the shooting victim (the barrister) being placed directly into an intensive care unit was considered. An example of the latter would be imagining a change in the rules in the triangle game or dot-clicking game. As noted above, such changes need to be treated with care, because they have the potential to substantially alter attributions of responsibility. Here again, proponents of the structural approach have argued that disputes about responsibility often do boil down to disputes about the correct or most appropriate causal model of the situation in question (Chockler & Halpern, 2004; Halpern & Hitchcock, 2010; Halpern & Pearl, 2005). This is another area that is ripe for psychological investigation. We suspect that people are adept at constructing causal models and counterfactual worlds that favor their own stake in a debate about responsibility.

### 4.4. Changes away from pivotality

According to the structural account an agent that is pivotal for the outcome is assigned full responsibility. However, there are situations where people systematically assign different degrees of responsibility to pivotal agents. We have already seen this in cases where agents differ in terms of their criticality. But there are also situations in which pivotal agents are equally criticality and yet receive differential attributions of responsibility. This is clearly shown in the results of the triangle game, where responsibility ratings were proportional to players' deviations scores even for players that all satisfy the same criterion. For example, consider the *Min* condition in the triangle game, where the team wins if at least one player gets the correct answer. On rounds where no one succeeds, all players are pivotal to the team loss, and thus according to the structural model each player is assigned full responsibility (*Resp* = 1). But the empirical data show that people are sensitive to how far each player is from the correct score, and assign responsibility in proportion to this deviation. A similar argument applies for the responsibility ratings for wins in the *Max* condition. Even though all players are pivotal for the win, those that deviate less from the correct answer are accorded greater responsibility for the win.

This suggests that intuitive judgments of responsibility can be graded even for pivotal causes. A natural extension to the structural model here is to consider changes *away from* pivotality as well as changes *to* pivotality. Thus, parallel to the notion that the responsibility assigned to an agent depends on the number of changes needed to make the agent pivotal for the outcome, one could add the condition that the responsibility assigned to pivotal agents depends on the number of changes needed to make them *non-pivotal* for the outcome. In particular, the more changes it takes to move an agent away from pivotality, the more blame they acquire for a loss or credit for a win.

This analysis is readily applied to the triangle game examples. In the *Min* condition, if player A deviates by 1 and player B deviates by 5, both are pivotal for the team's loss (changing either player to 0 would switch the loss to a win), but player A requires fewer changes (in deviation points) than player B to make him non-pivotal for the loss. Thus, A is attributed less responsibility for the loss than B (which fits with the empirical data). Similarly, in the *Max* condition, suppose all players deviate by less than 3, so all are pivotal for the win. If player A deviates by 1, and player B by 2, then less changes are required to make B non-pivotal. Thus, B is assigned less responsibility for the win (which fits the empirical data).

Furthermore, we also see evidence for this proposal in the data of the novel experiment. Consider, for example, the set of challenges shown in Fig. 10C. *A*'s criticality and pivotality is the same in situations 2–4. However, participants blamed A significantly more for the loss in situation 4 (*M* = 95.08, *SD* = 11.89) in which three changes away from pivotality were required compared to, for example, situation 2 in which only one change would have rendered *A* non-pivotal (*M* = 84.05, SD = 21.6), *t*(39), = 2.94, *p* = .006, *r* = .18. Consider the set of challenges shown in Fig. 10D and compare the credit that *A* received in situation 2 versus situation 4. *A* is fully critical and pivotal in both situations. However, participants attribute significantly more credit to *A* in situation 4 (*M =* 83.78, *SD* = 23.44) compared to situation 2 (*M =* 72.30, *SD* = 27.87), *t*(39), = 4.75, *p* < .001, *r* = .37. This effect is not predicted by the new responsibility model but can be explained by using the notion of robust pivotality. Whereas only one change would already render *A* non-pivotal in situation 2 (i.e., changing *B*), three changes are required to render *A* non-pivotal in situation 4.

The notion of “changes away from pivotality” seems a viable extension to the responsibility model, and will be pursued in future work. It also meshes with the idea that a causal relation can be assessed in terms of its robustness (or insensitivity) to external influences and circumstances (Woodward, 2006).

### 4.5. Changes as causes

One potential shortcoming of the structural definition of actual causation is that it seems to neglect an important aspect of actual causation—the sense in which a cause is often a change in state (rather than just an instantiation of a variable). Thus, Glymour et al. (2010) argue that the definitions of actual cause given by Halpern and Pearl (2005) and Woodward (2003) are overly static and do not accommodate the fact that “we tend to think of causes as changes, or happenings.”^17^ For instance, Halpern and Pearl explicitly state that “events” on their account correspond to sets of possible worlds rather than changes in states. For them, therefore, actual causation is a relation between static states (variable values), and changes in states are only introduced in the counterfactual part of their analysis. Glymour et al. argue that this leaves out the critical notion of a change in state, and they point out that actual cause judgments can be sensitive to the prior state of the system (such that a causal model with an identical set of variable values can lead to different actual cause judgments depending on which state preceded it). Glymour et al. maintain that these shortcomings can be dealt with by viewing the causal model framework from a different perspective, one based on state transitions and taking into account prior system states.

We agree that the notion of state change is often crucial to judgments of actual causation, and that the current family of structural models does not capture this in a natural way (cf. Gerstenberg, Goodman, Lagnado, & Tenenbaum, 2012). But as Glymour et al. point out, a different perspective on the causal model framework, which takes state changes (and prior system states) as basic components, can readily accommodate this. Indeed, this suggests a novel line of psychological research where prior system states are varied while holding constant the actual model and variable values. Along with Glymour et al.'s intuitions, we expect people to be likewise sensitive to these initial states. All of the studies reported in this article have a default starting state for the system (before anyone does anything), so the potential dependence on prior states does not present an issue. However, future studies could explore this interesting factor.

## 5. Conclusions

This article has explored the structural model account of causal responsibility, with particular focus on simple multiple agent games. We have proposed a novel model of intuitive attribution that takes into account both backward- and forward-looking aspects of responsibility. Given a causal model of the situation, and knowledge about the actions of the agents and the outcome, people look backward to see whether an agent was pivotal (or close to pivotal) for the outcome. But they also factor in how critical the agents were in the first place, before these actions were taken. This combination of retrospective and prospective responsibility is intuitively plausible and captures participants' judgments in our empirical studies. It also parallels legal conceptions of responsibility, where theorists distinguish retrospective (historic) and prospective functions of legal responsibility (Cane, 2002). Indeed, Cane (2002) argues that the primary function of the law is to “tell us what are responsibilities are” (prospective responsibility), and that holding people accountable for their failures to do this (retrospective responsibility) is a secondary concern (Cane, 2002, p. 63). We speculate that intuitive judgments of responsibility might also be best understood in terms of forward-looking goals, with the retributional aspect serving as a means to this end.

Highlighting the prospective as well as retrospective components of responsibility attributions also cements the central role played by causal models—which serve both a strong predictive purpose, and an explanatory and attributional role in human cognition. The causal model framework, however it is characterized in detail, is ideally suited to this role. It allows us to predict the consequences of future actions, as well as to identify the causes of outcomes that have already occurred.

As noted in the article, there are various factors that the structural model approaches, in their current formulation, do not cover. There are also potential limitations with their specific definitions of actual cause, and perhaps with the search for any definition tied to necessary and sufficient conditions. Irrespective of these challenges we believe that the causal model approach furnishes us with a flexible framework to explore both formal and psychological theories of causal attribution. Indeed, there is a fruitful interplay between the formal developments and the empirical data on people's intuitive judgments. The process of discovering a principled equilibrium between formal and empirical accounts is underway.

## Notes

1 This is a real case that took place in London, May 6, 2008. For details of the inquest, see: http://www.ipcc.gov.uk/documents/investigation_commissioner_reports/saunders-21-12-10.pdf

2 Legal theorists and practitioners are aware of the shortcomings of this definition, and there are further requirements for causality in law (Moore, 2009).

3 Here, variables can refer to properties, event types, or propositions. For our current purposes, we can sidestep philosophical questions about the exact nature of the causal relata, although this does raise some important issues when considering actual causation. A key issue is the interpretation of an event (whether in terms of a set of possible worlds or a transition between states). This is discussed in more detail later in the article.

4 This is not intended as a rigorous definition—see Pearl (/2009^2000^) and Woodward (2003) for more explicit definitions.

5 This sounds circular (i.e., that we are assuming what we want to show), but note that so far we are just setting up the general-level causal model and have made no claims about the values of the variables or ascriptions of actual causality. However, it does highlight that the structural model approach is not offering a reductive account of causality but rather representing our causal claims. Also, note that the coroner in the siege case is making a judgment about the appropriate form of the causal function to apply in that case, and from that the actual cause assignments follow. He is not simply evaluating, but looking at the causal (physiological) processes that led to the barrister's death.

6 See Sloman and Lagnado (2005) for empirical data showing that people endorse such reasoning.

7 On the structural model account, an event (or state of affairs) corresponds to the assignment of a value to a variable, for example, BA = 1 represents that the barrister aims his shotgun at police. According to Halpern and Pearl (2005), an event is a set of possible worlds (rather than a change from one state of affairs to another). Thus, for them actual causation concerns a relation between one set of variable values (the causes) and another set (the effects).

8 Here we define “pivotality” in terms of counterfactual dependence: “*c* is pivotal for *e*” is equivalent to “*e* counterfactually depends on *c*.”

9 The CH account is not restricted to human agents; it covers multiple causes more generally (e.g., it is applied to fault diagnosis in complex computer systems). However, we will focus on human agents in this article.

10 It is worth noting that a simple alternative account of the voting situation would be the diffusion of responsibility, because fewer members vote for A in the 6–5 case, and perhaps there is a “fixed” amount of responsibility to be shared. We shall see later that this by itself does not explain people's patterns of attribution. Also, it seems to be undermined by comparing a 6–5 outcome with a 3–0 outcome (given a committee of three). A simple “fixed sum” diffusion model would give members in the 3–0 vote a higher degree of responsibility, but the CH model would give a higher degree to members in the 6–5 case.

11 Note that conjunction and disjunction are counterparts in the sense that a game that has a conjunctive function for wins has a disjunctive function for losses, and *vice versa*. For example, if a conjunction of passes is required for the team to win, then a single fail is sufficient for the team to lose. Likewise, if a single pass is sufficient for a team to win (disjunction), then a conjunction of fails incurs a team loss.

12 The failure of player D ensured that manipulating the individual outcomes of the other players between conditions never renders any of them pivotal.

13 It should be noted that the heuristic model does not readily extend to more complex structures; therefore, a more sophisticated model, such as the probabilistic model mentioned here, is needed for full generality. However, for present purposes the key point is that retrospective attributions appear to depend on criticality as well as pivotality.

14 A demo of the experiment can be accessed here: http://www.ucl.ac.uk/lagnado-lab/experiments/demos/structure_demo.html.

15 This is strictly true in some sets of challenges, whereas others exhibit some correlation between criticality (as predicted by the *heuristic model*) and pivotality (as predicted by the *structural model*). The overall correlation for the 36 challenges used for example, in the experiment was low with *r* = .29.

16 For the model predictions of the *critical-pivotality model* in Figs. 8, 9, and 10 we used the heuristic as a model of criticality and the structural model as a model of pivotality. Furthermore, we allowed *w* to vary between the different sets of challenges.

17 A similar point is made by Steward (2010) when discussing the ontology of causation, where she distinguishes three categories of cause: “makers-happen,” “matterers,” and “movers.” The structural definition of Halpern and Pearl (2005) focuses on “matterers,” whereas the notion of change or happening proposed by Glymour et al. corresponds to “makers-happen” in Steward's taxonomy.
